# GOATEA: gene set enrichment analysis in R with shiny interactive visualizations

**DOI:** 10.1186/s12859-026-06541-w

**Published:** 2026-06-18

**Authors:** Maurits A. W. Unkel, Jeff A. Beeler, Steven A. Kushner, Femke M. S. de Vrij

**Affiliations:** 1 Department of Psychiatry, Erasmus MC University Medical Center, Rotterdam, The Netherlands; 2 Department of Psychology, Queens College, City University of New York (CUNY), Flushing, NY USA; 3 CUNY Neuroscience Collaborative, Biology and Psychology Programs, The Graduate Center, City University of New York (CUNY), New York, NY USA; 4 Stavros Niarchos Foundation (SNF) Center for Precision Psychiatry & Mental Health, Columbia University, New York, NY USA; 5 Department of Psychiatry, Columbia University Irving Medical Center, New York, NY USA; 6 Erasmus MC Center of Expertise for Neurodevelopmental Disorders (ENCORE) , Erasmus MC University Medical Center, Rotterdam, The Netherlands

**Keywords:** GSEA, GOAT, GOATEA, Genomics, Proteomics, Bioinformatics, R, Shiny, Interactive, Visualization

## Abstract

**Background:**

High-throughput genomic and proteomic technologies are used to study biological systems by performing differential expression analysis across various experimental conditions. Geneset Ordinal Association Test (GOAT) is an analytic method recently introduced to statistically evaluate the differential expression of a defined set of genes or proteins. Despite the availability of numerous enrichment tools, many lack accessibility for users without programming expertise, provide limited continuation beyond listing top enriched terms, and offer little support for interactive visual exploration or hypothesis generation. Moreover, existing web-based platforms rarely support multi-contrast comparisons and generally omit gene-level or network-based context for pathway analysis.

**Results:**

To address these limitations, we present Geneset Ordinal Association Test Enrichment Analysis (GOATEA), an R/Shiny application that implements and extends the GOAT algorithm with interactive visualization, multi-contrast comparison, and integrated gene- and network-based context for bottom-up pathway analysis, enabling comprehensive enrichment analysis.

GOATEA supports independent analysis of transcriptomic and proteomic data. To demonstrate its capability to integrate matched modalities, we applied it to the Colameo dataset containing paired mass spectrometry and RNA sequencing data. This proof-of-concept example highlights the tool’s strength in enabling multi-omics analyses and simultaneous comparison of multiple contrasts.

An interactive overlap analysis identified 458 shared genes for focused enrichment and network exploration. By integrating these results in a gene- and network-based context for bottom-up pathway analysis, GOATEA applies a stringent interaction confidence threshold to emphasize qualitative protein–protein interactions, highlighting topic-relevant associations for further hypothesis generation.

**Conclusions:**

GOATEA streamlines enrichment analysis workflows by combining the GOAT algorithm with interactive visualizations in a user-friendly graphical interface. It facilitates exploratory analysis and hypothesis generation for researchers with or without programming expertise. GOATEA is available as an open-source tool, with full documentation, including usage vignettes (https://mauritsunkel.github.io/goatea/).

**Supplementary Information:**

The online version contains supplementary material available at https://doi.org/10.1186/s12859-026-06541-w.

## Background

High-throughput genomic and proteomic technologies are used to study biological systems by performing differential expression (DE) analysis across various experimental conditions. This generates measured effect sizes and corresponding statistical values for each independent transcript or protein, which is frequently on the order of 10^3^–10^5^ depending on the nature of the experiment being performed. The resulting datasets are high-dimensional and statistically complex, necessitating computational methods for systematic and unbiased interpretation of differential expression patterns to investigate and visualize biological mechanisms.

Gene set enrichment analysis (GSEA), also known as functional enrichment, is a statistical method for testing whether genes or proteins are enriched in gene sets related to specific biological aspects of interest [1]. Among the most widely used annotation frameworks, the Gene Ontology (GO) consortium classifies genes into hierarchically structured categories describing biological processes (GOBP), molecular functions (GOMF), and cellular components (GOCC) [2]. Beyond GO, numerous other gene set databases exist, each tailored to specific organisms or biological aspects such as pathways or diseases, further expanding the scope of functional enrichment analyses,

With over-representation analysis (ORA), a type of GSEA, researchers can investigate whether predefined sets of genes are overrepresented in gene sets. Recently, gene set ordinal association test (GOAT), a parameter-free algorithm for GSEA of pre-ranked gene lists, was introduced [2]. GOAT precomputes null distributions from standardized gene scores, enabling enrichment testing with very short runtimes (~1 s). Validation on simulated benchmarking data demonstrated invariance to gene list and gene set sizes with estimated p-values calibrated under the null hypothesis. GOAT identified a higher number of significant gene sets compared to conventional ORA methods. To assess whether this reflected true sensitivity rather than false-positive inflation, null and foreground gene sets were simulated and evaluated using receiver operating characteristic analysis [2]. GOAT successfully differentiated between enriched (foreground) and uniformly sampled (null) gene sets, achieving near-perfect separation with a 5% partial area under the curve at 95% specificity, thereby confirming improved sensitivity while maintaining specificity.

Generally, the top significant gene sets resulting from ORA methods are summarized in dot or bar plots, often overlooking additional relevant complementary insights to be obtained from deeper exploration.

Here, we describe GOATEA, an R Shiny application and package that facilitates analysis and interactive visualization of ORA methods performed by GOAT. The GOATEA Shiny interface integrates both common and novel visualizations. These include pre-enrichment plots such as *EnhancedVolcano* [3] and *UpSetJS* [4], post-enrichment representations including dot plots and semantic similarity trees [5], as well as novel InteractiveComplexHeatmaps for term- and sample-symbol overviews. Finally, an interactive STRING [6] protein-protein interactions (PPI) network igraph is provided for bottom-up network-level analysis. These interactive visualizations allow users to explore data and interpret enrichment patterns within a gene- and network-based context for bottom-up pathway analysis, enabling network-guided hypothesis generation.

Different exploration types exist to aid in the investigation of enriched gene sets, many of them are dispersed across varying code packages and tools, often without an integrated approach to identify related sets and relevant subsets or individual genes. In Supplementary Table 1 a qualitative comparison shows traditional (GSEA [1], DAVID [7]), similar (GOAT [2], ShinyGO [8], FLAME [9]) and other commonly (Enrichr [10], g:Profiler [11], ClusterProfiler [12], Metascape [13]) used tools and packages.

GOATEA offers a robust and flexible framework for functional enrichment by leveraging the capabilities of the GOAT R package. Through this integration, GOATEA supports the GOAT algorithm alongside more traditional strategies that are integrated into the GOAT package, such as ORA and the GSEA protocol established by Subramanian et al. (2005). Additionally, GOATEA and GOAT are the only tools that can utilize either preranked (GSEA) or unranked (ORA) symbol lists with either including or excluding effect sizes and *p*-values respectively.

GOATEA is also most flexible in interface options, providing easy web tool access via HuggingFace (https://huggingface.co/spaces/Mausaya/GOATEA), local R Shiny GUI for more power, local R package for reproduction and Docker for a robust and deployable option. Most tools provide only static visualizations or allow for aesthetic adjustments. Some tools give informative interactivity, such as clicking to sort, mouse-hovering for information showing and selecting for database linkage. Additionally, most tools that provide informative interactivity also enable reparameterization of thresholds and recreate the plots on the fly. GOATEA and FLAME are the only tools that also allow for additional exploration through their interactivity, selecting gene sets or genes and using these for visual exploration or re-analysis. More broadly, existing enrichment tools frequently offer little support for deep explorative data interaction that incorporates network-based context for pathway analysis. Moreover, single-list contrast input is generally outdated, as most tools, including GOATEA, offer multi-list or batch input. In this study, a contrast is defined as a pairwise comparison between samples representing different experimental conditions, from which differentially expressed genes or proteins are derived. With multi-list input there is an increasing need for enrichment speed. Classic GSEA is slow and can take minutes or more, fast GSEA and ORA methods are in the range of seconds or less, GOATEA uses GOAT with precomputed data distributions and runs in under a second. Other tools offer a plethora of visualizations, some of which are not included or focused on in GOATEA. However, only GOATEA and FLAME offer pre-enrichment visualizations (Volcano, UpSet), enabling an integrated workflow of full symbol list exploration from quality control to functional enrichment. ClusterProfiler and Metascape do offer an UpSet and Circos plot respectively, although these are available post-analysis. Most tools, including GOATEA, have published manuscripts, web documentation and step-by-step tutorials or vignettes. All tools provide access to a multitude of organisms with GOATEA providing access to key model organisms. Similarly, most tools support a broad range of gene set databases, including the traditional gold standard MSigDB [1], the commonly used Gene Ontology [14] as well as pathway, disease or other niche databases. GOATEA supports the .GMT file format, that allows users to upload the downloaded database exports. GOATEA addresses key limitations of existing enrichment tools by providing accessibility for users without programming expertise, streamlining integrated enrichment workflows and empowering comprehensive, interpretable, and reproducible functional analyses.

## Implementation

### Development and installation

GOATEA was written in the R programming language (v4.5.0) [15], utilizing *GOAT* (v1.1.2, 3) for the enrichment analyses and using *shiny* (v1.10.0) [16] and *shinydashboard* (v0.7.3) [17] for the graphical user interface (GUI). GOATEA is platform agnostic and is available as an open-source Shiny application within an R package available on Bioconductor (v3.22, https://bioconductor.org/packages/devel/bioc/html/goatea.html) and hosted on GitHub (https://github.com/mauritsunkel/goatea), where installation and usage are detailed in the accompanying documentation. Development version 0.99.8 was used for the analyses and visualization showcase the demonstration provided in this paper. Users are recommended to use the latest (development) version, as synchronized on GitHub, Bioconductor and Docker (as used in HuggingFace). The GUI uses custom and *shinyjs* (v2.1.0) [18] for JavaScript background functionality with shiny elements and *shinyjqui* (v0.4.1) [19] for JavaScript queries on UI interactions and effects for shiny. In addition, *htmltools* (v0.5.8.1) [20] were used to supplement the GUI with html elements and classes. In the background, the following Tidyverse [21] packages were used for structuring and efficiency of code: *dplyr* (v1.1.4) [22], *tidyr* (v1.31) [23], *purrr* v(1.2.0) [24], *tibble* (v3.3.0) [25] and *ggplot2* v(4.0.0) [26]. Reading and writing tabular data was done with *openxlsx* (v4.2.7.1) [27].

On minimal system requirements, GOATEA is platform independent. A standard office computer with 8GB RAM and Intel Core i5 CPU or later equivalent is more than sufficient. Very little storage space is required as outputs are only plots and tables. Development and testing were performed on an Alienware Area-51m laptop with Windows 11 64-bit, 64GB RAM and Intel Core i7 CPU. Analysis script with automated plotting runs within minutes. GUI steps run within seconds to minutes as well, mostly dependent on loaded gene set size. Handling larger datasets will mainly depend on the number of samples, requiring more manual input in the GUI. Only exceptionally large gene set data will require more computing power. If users run into trouble they are advised to create an issue on GitHub (https://github.com/mauritsunkel/goatea/issues).

### Datasets and databases

The GOAT package contains downloadable gene expression and proteomics data. Input gene- and protein data are referred to as data lists. Here, GOATEA’s UI is showcased using the RNA transcriptomics and mass-spectrometry (MS) proteomics data from Colameo et al [28], This and other prepared data can be obtained via *goat::download_goat_manuscript_data(),* downloaded via GitHub (https://github.com/mauritsunkel/goatea/tree/main/data) and HuggingFace and is showcased in a vignette *(*https://mauritsunkel.github.io/goatea/*)* as this dataset highlights GOATEA’s multi-omics and multi-contrast comparison capabilities and was previously used to benchmark the underlying GOAT algorithm. The GOAT enrichment data are made available as tables (.xlsx) in Additional File 1. They described how synaptic scaling, a form of homeostatic plasticity, was regulated at the molecular level within different subcellular compartments of primary rat hippocampal neurons. Although rat tissue was used (*Rattus Norvegicus*), gene and protein names, which we will henceforth denote as symbols, were mapped to NCBI Entrez identifiers of *Homo Sapiens* by GOAT as described in its manuscript, to homogenize all symbols for benchmarking the enrichment algorithm. On the GOAT web platform (https://ftwkoopmans.github.io/goat), users can find the 'gene ID mapping' tool available with documentation. There, only Human Entrez (NCBI) gene identifiers are currently supported to convert by providing gene symbols in the 'symbol' column of a .xlsx/.csv/.tsv formatted file. This is a good way to see how many symbols can be successfully converted. GOATEA supports symbol conversion for all supported organisms via the user specified 'org.Xx.eg.db'. If GOATEA is used from R, the user will additionally see a percentage of properly converted symbols. GOAT defined significance of the symbols by setting a stringent *p*-value threshold of 10^−4^, accounting for the large number of significant hits. This yielded 11921 genes and 3934 proteins of which 2684 and 1494 were significantly different, respectively. To enable visual comparison, MS effect sizes were linearly scaled down to the RNA effect sizes with *goatea::scale_values_between()*.

Protein-protein interaction (PPI) data was obtained from the STRING database [6]. The GUI allows users to specify the latest or any available STRING database versions, which can be checked through *goatea::get_string_ppi()*.

GO gene sets were downloadable, pulling organism specific annotation data via *AnnotationDbi* [29]. These organisms were made available in the current version of GOAT and GOATEA: *Homo Sapiens* (Human), *Mus Musculus* (Mouse), *Rattus Norvegicus* (Rat), *Pan troglodytes* (Chimpanzee), *Macaca mulatta* (Rhesus Monkey), *Danio rerio* (Zebrafish), *Drosophila melanogaster* (Fruit Fly) and *Caenorhabditis elegans* (Worm).

### Workflow and use

**Fig. 1:**
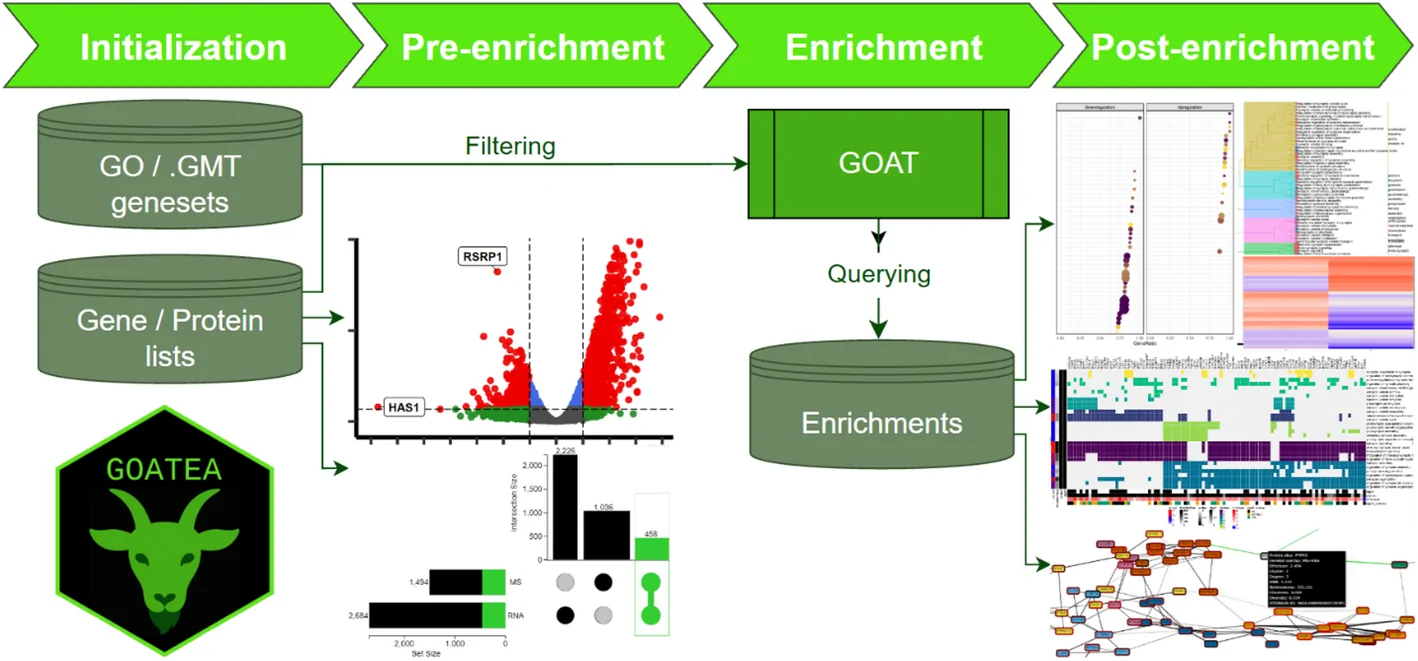
GOATEA workflow. Showing initialization, loading symbol (gene and protein) lists in parallel with gene sets (e.g. GO, .gmt file). Pre-enrichment visualizations of the loaded data with volcano and overlap plots. Filtering the symbol lists and gene set data together before GOAT enrichment. Then querying the enriched gene sets and performing enrichment analysis through post-enrichment visualizations with dotplots, treeplots, interactive overview and comparison heatmaps as well as protein-protein interaction networks. Cylinders = data. Rectangle = process. Plain text = task.

**Fig. 2:**
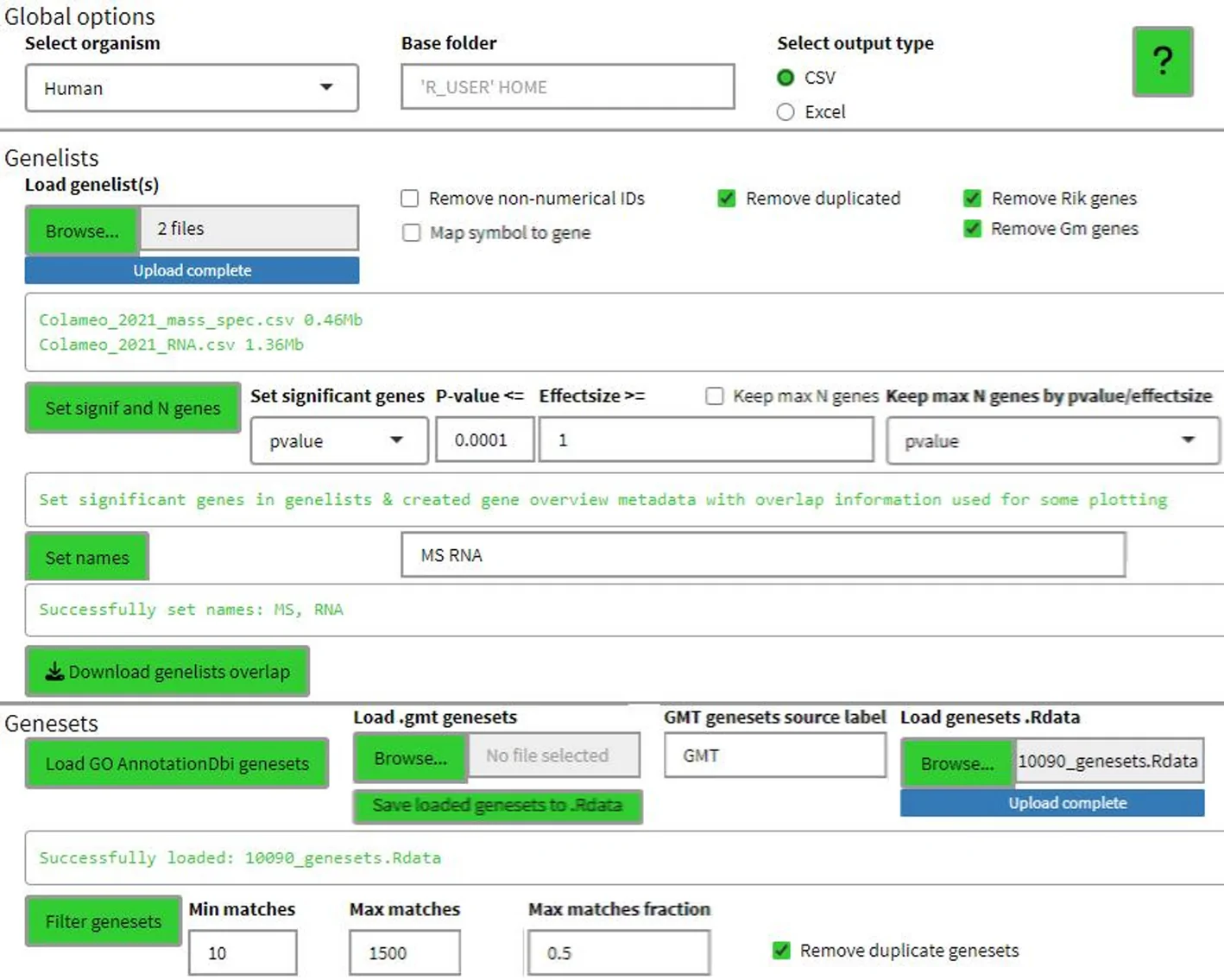
Cropped GUI screenshot showing the initialization landing page in GOATEA. Global parameters are set here and data is loaded and filtered. Status messages are shown to the user, on success the subsequent buttons become available. UI elements are cursor-hoverable to view pop-up documentation.

The overall workflow for GOATEA is depicted in Fig. 1. Figure 2 presents the GUI, which guides the user through data input, enrichment and visualization. Subsequent figures show the visualizations created within the UI. Users can manually run the GUI as a local Shiny application or access it through their web browser. Running the GUI as a Shiny application is generally recommended, as browsers restrict users from specifying download locations on their system. HuggingFace provides the easiest access to GOATEA as a direct web tool. Note that runtimes here were slower, specifically for loading large datasets like the GO annotations via AnnotationDbi, which could take up to minutes. In all cases, as long as the loader animation was running, the application was working on the background. For technical users, scripted analyses and visualizations were made available through exported package functions to build automated workflows. Example workflow vignettes were made available on GitHub (https://mauritsunkel.github.io/goatea/). Additional usage instructions can be found in the local R package documentation.

As a customization, the user can globally set the GUI colors of a predefined grouping of UI elements before initialization on the local R version. On GUI startup, the user lands on the initialization tab. All UI elements can be cursor-hovered to view a descriptive overlay to aid the user. Each tab has a ‘?’ button that opens a modal dialogue overlay with a tab description to provide documentation to the user. Every tab has ‘go to’ navigation buttons for user-guidance and quick switching between tabs can also be accomplished from the tab menu. Some buttons only become available once previous steps are successfully performed to effectively lead the user through the GUI. All tables and figures are exportable.

### Gene set enrichment analysis with GOAT

In GOATEA, varying comparative gene set enrichment analysis methods were available as implemented by GOAT. This facilitated ease of comparison and benchmarking using the same codebase and flow. Different scoring types were available. Users could select effect size as the default or choose p-value. When effect size was selected, both negative and positive effect sizes were considered by default. Otherwise, users could select from only positive (up), only negative (down) or absolute effect sizes. This non-mixed effect size selection transformed or filtered effect sizes before performing GOAT. Bonferroni and Benjamini-Hochberg were available as methods for p-value adjustment, and the number of gene sets was used for multiple testing correction. Additionally, p-adjusted cutoff and minimum number of significant symbols could be given as thresholds on significance for enrichment results. By default, GOAT enables enrichment of maximally 20.000 symbols, ordered by absolute effect size or least significant p-values, when using the precomputed null distributions. The bootstrapped version of GOAT had no maximum number of symbols. After running GOAT, the enrichment result could be filtered by querying terms and symbols based on their name and features.

### Shiny interactive visualization for data exploration

Pre-enrichment, volcano plots were created with *EnhancedVolcano* (v1.22.0) and made interactive with *plotly* (v4.10.4) [30]. The interactive UpSet overlap plot was made with *UpSetJS* (v1.11.1). UpSet plot offers the intersect, distinct and union modes for overlapping and then selecting symbols which are taken along for downstream heatmap visualizations and PPI network plotting. Intersection are symbols present in all selected contrasts, to visualize a shared core. Distinct symbols are unique to specific intersections, to visualize exclusive signatures. Union symbols are all symbols found in any of the selected contrasts, to visualize a comprehensive overview. The enrichment table view is a DataTables object, a wrapper of the JavaScript library *DT* (v0.33) [31]*.* Post-enrichment, a *ggplot2* (v3.5.1) based split-dot plot and a term tree plot as implemented in *enrichplot* (v1.24.4) show top enrichment. The overview symbol-term and sample-symbol heatmaps were created with *ComplexHeatmap* (v2.24.0) [32] and made interactive with *InteractiveComplexHeatmap* (v1.12.0) [33]*.* For the PPI network graph, temporary downloaded data was converted to columnar Parquet format using *arrow* (v18.1.0.1) [34]*.* Then the data was formatted for input into *igraph* (v2.1.4) [35] using its structure to create the final PPI graph with *visNetwork* (v2.1.2) [36].

### Demonstration and discussion

A showcase is presented to demonstrate GOATEA’s utility in exploring and comparing genomics and proteomics data for generating hypotheses, as well as giving an overview of its user interface. For this purpose, the Colameo et al. (2021) dataset is used, which is particularly well-suited for benchmarking GOATEA because it contains paired RNA transcriptomic and mass spectrometry (MS) proteomic data collected from the same experimental conditions. The study investigates homeostatic synaptic plasticity, a fundamental mechanism by which neurons maintain stable activity despite perturbations. This paired design enables direct comparison between gene expression and protein abundance, making it ideal for showcasing GOATEA’s integrative analysis capabilities.

The GOATEA UI is split into 9 tabs within 4 tab groups, loading data within the initialization tab group functions as the landing page, with pre-enrichment plotting, enrichment and post-enrichment visualization functioning as the bulk of the enrichment analysis and exploration. The landing page includes the global parameterization for GOATEA as well as options for loading and filtering the data lists and the gene sets (Fig. 2).

### GOATEA initialization and data processing

On initialization, the user can set global options and load and filter the data. For the global options, the user selects one of the available organisms. Here, ‘Human’ was selected, as the Colameo rat data were mapped to the human genome by GOAT to enable benchmarking datasets of different species. A user-set destination folder, by default the R user home directory, will be used for exporting outputs. If the selected folder is unavailable, the system download folder is used as the default, similar to web browser usage. Additionally, the user specifies an output format for resulting tabular output to Excel or comma-separated values.

Users can upload one or multiple data lists representing contrasts. Uploading multiple lists enables overlapping and visually comparing the data, as well as adding metadata to the output. Data lists were expected to be in the GOAT input format as specified in the documentation. Short sample names can be set for individual data lists. Here, we set names to MS and RNA for visual clarity.

For ease of use, GOATEA provides handling input data without p-values, effect sizes or gene identifiers. If symbols are present, the user can choose to map these to Entrez identifiers via *AnnotationDbi::mapIds()*. GOAT requires these features to be present and to be assigned significance, which GOATEA performs through user-provided thresholds for p-value, effect size or both. By default, duplicated gene symbols were removed. Significance by default is set to p-value <= 0.05 and effect size >= 1. Here, we mimic significance defined in Colameo by using only the p-value <= 0.0001 threshold.

Next to loading the data lists, gene set data can be obtained in many ways. GO gene sets were downloaded via *goat::load_genesets_go_bioconductor()* to obtain the human-specific data annotation package *org.Hs.eg.db* [37], saved to .rdata and loaded locally. GOATEA supports loading from many sources through a commonly used .gmt (generic mapping tools) file format, available via both the GUI and R version utilizing the *goat::load_genesets_gmtfile()* function. Downloading gene set data is possible via web services like MSigDB[1] and Enrichr [10], these and others also provide application programming interfaces (APIs) or web databases to download from. Additionally, users can access gene sets through GOAT itself via the *goat::download_genesets_goatrepo()* function, load the most recent GO annotations directly using *goat::load_genesets_go_fromfile()* with gene2go and OBO files instead of waiting for scheduled updates in GOAT or Bioconductor organism packages, or obtain synapse-specific gene sets from the SynGO, created by the developer of GOAT, via *goat::load_genesets_syngo(*[38]*) (*https://syngoportal.org/*)*.

To make ready for enrichment and visualization, data and gene sets were filtered with default parameters to remove duplicates gene sets and to retain gene sets matching between 10 and 1500 symbols. For every step, the user can see status information displayed in the text areas. On successful processing, the go-to-buttons become available to lead the user to the next steps. In this case, pre-enrichment interactive volcano and UpSet overlap plotting of the loaded data lists.

### Pre-enrichment visualization using EnhancedVolcano and UpSet plots

**Fig. 3:**
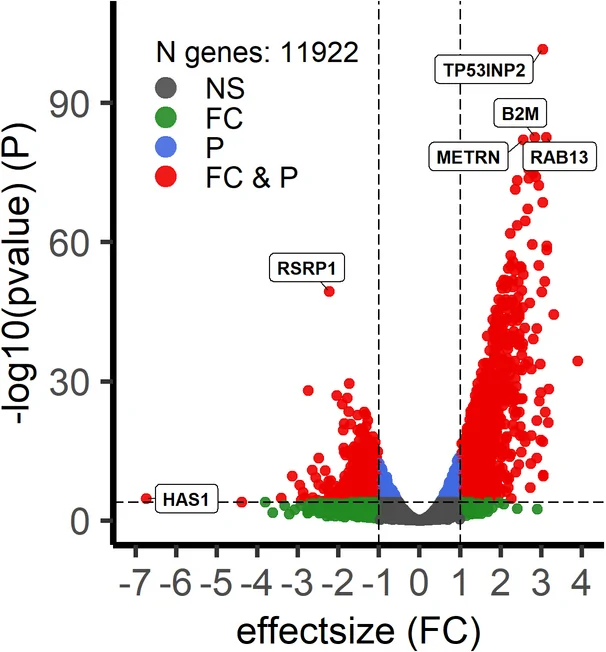
Pre-enrichment interactive EnhancedVolcano plot. The effect size (log2 fold-change) is plotted against the -log10 formatted p-value. Labels are added to the top significant symbols. Legend shows the total number of symbols and colors display symbol type. FC = fold-change (effect size), P = *p*-value, NS = non-significant.

The pre-enrichment plots are optional steps in the GOATEA workflow. The interactive EnhancedVolcano plots can be made of a selected data list. Here, the RNA data list is selected and the plot shows symbol significance by effect sizes versus p-values with user-set thresholds with labels on top significant symbols (Fig. 3). On hovering over a symbol, plotly shows overlay information. By selecting symbols by dragging, they can be added to the selection.

**Fig. 4:**
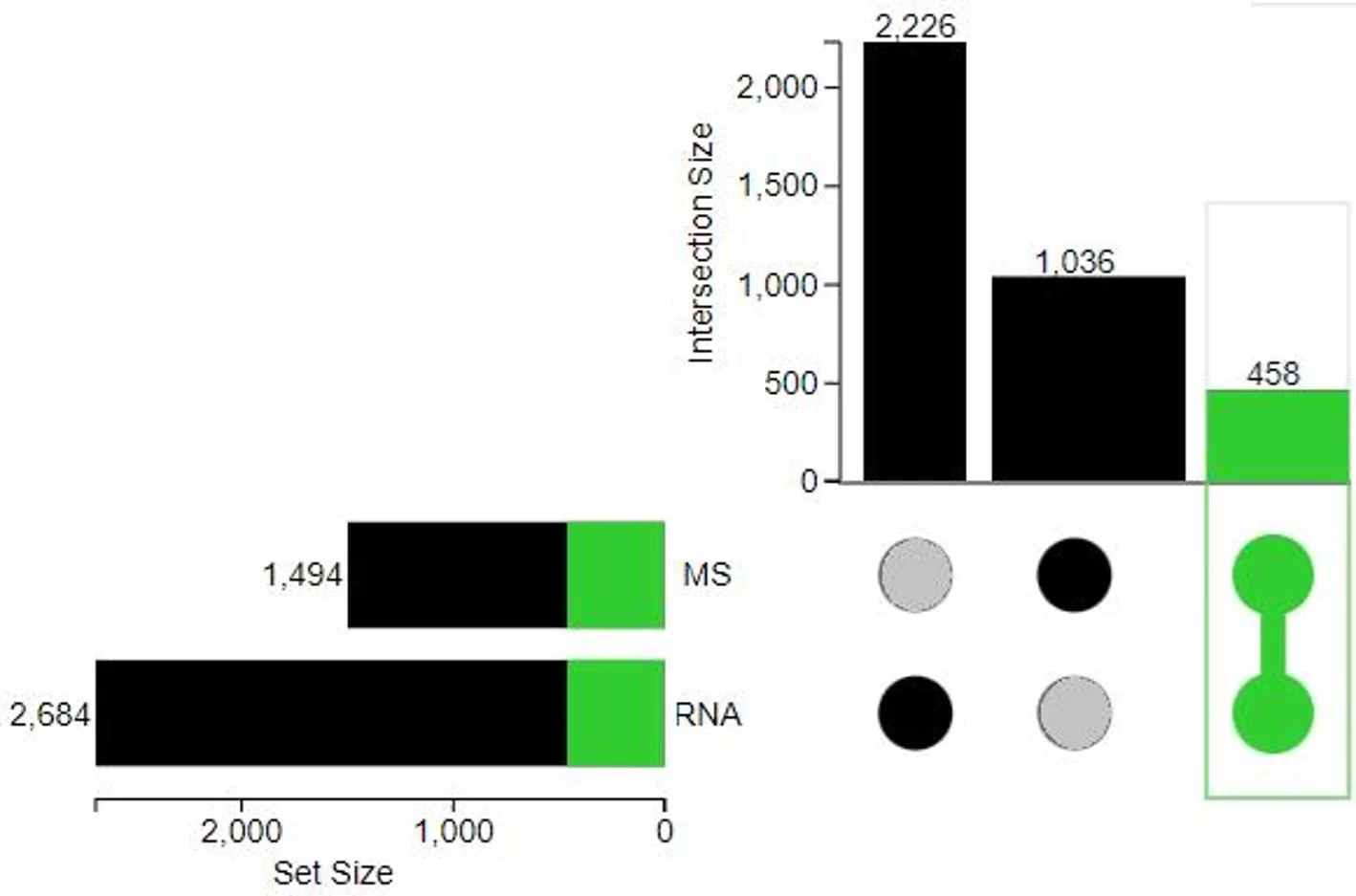
UpSet interactive overlap plot. On the left, histograms show set size of the samples. On the right, histograms show the intersection size of the number of symbols between intersections of the samples. Overlapping sections are displayed by dots and connections between them beneath the histograms. On hover, the set is colored and on selection it remains colored, allowing the user to add the symbols to the selection.

The interactive UpSet overlap plot shows the number of significant symbols between data lists for the user to make selections on for downstream visualizations (Fig. 4).

The user can choose between three overlap modes: distinct as default, union and intersect. Here the distinct overlap mode is selected and 458 symbols overlapping in both the MS and RNA data lists were selected for downstream exploration, enabling comparison of these data. At any point, the user can continue to enrichment with the go-to buttons.

### Gene set enrichment analysis using GOAT

**Fig. 5:**
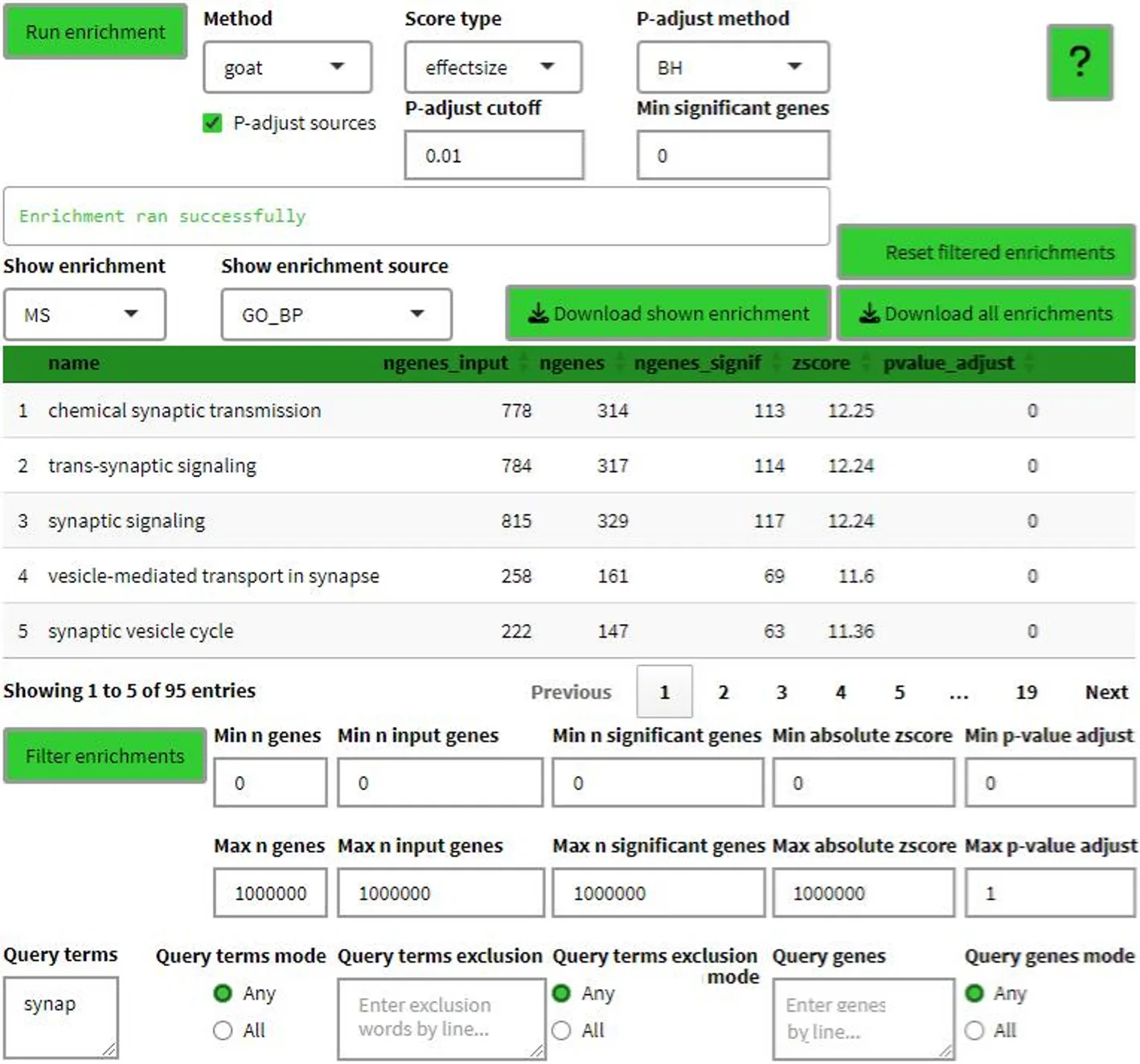
Cropped GUI screenshot of the gene set enrichment parameters, the resulting enrichment showing specific features and the dynamic querying filters.

The main data analysis step in GOATEA is GOAT, which performs over-representation analysis on the parallel filtered symbols and gene sets. UI for the enrichment plus its querying and filtering is shown in Fig. 5.

Effect size is used as the score type to take the differences in biological expression into account. Benjamini-Hochberg p-value adjustment is used for most stringent testing of significant gene sets at the default cutoff p-value <= 0.01. No pre-filtering of minimum significant symbols is needed as possible with post-hoc querying. Enrichments results are stored in a DataTables object in the background for data manipulation and storing in local memory to be shown to the user as a table with specific filterable features. Numerical features can be filtered by supplying a minimum and maximum value. The term and symbol names can be queried by text. Query modes provide the user with flexibility in matching term names or groups of genes partially or exactly. For our use case, ‘synap’ is used as query term to match by regular expression with term names that include ‘synapse’ and ‘synaptic’ to hit relevant gene sets for the Colameo data exploration. After the enrichment runs successfully, the go-to-buttons for the post-enrichment plotting become available.

#### Post-enrichment visualization using ComplexHeatmaps and STRING network igraph

**Fig. 6:**
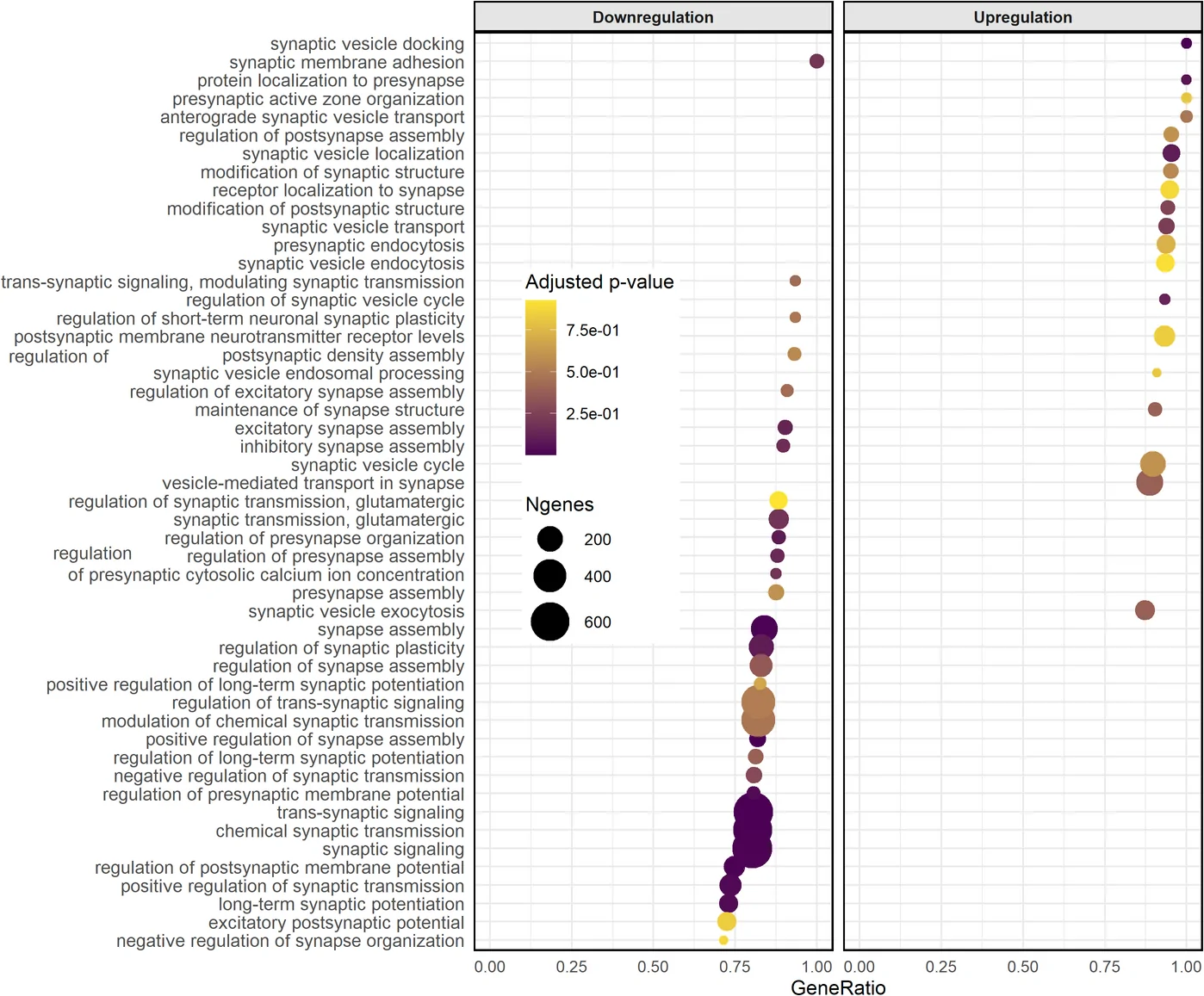
Dot-plot of top enriched terms split by up- and down regulation. Terms are sized by the number of symbols in their gene set and colored by the adjusted *p*-value. Gene ratio is the number of input genes matching the term divided by the number of total genes in the term.

Post-enrichment visualizations are based on the current DataTable view on the enrichment tab. A split-dot plot (Fig. 6) can be made for the set number of top enriched terms, by default 50. This dot plot shows the terms split by down- and upregulation, as obtained from the effect sizes differences of the symbols, and colored by the adjusted *p*-value. Gene ratio is the number of input genes, which is the dot size, matching the gene set divided by the number of total genes in the gene set. Here we can see that synaptic signaling is a broad term in the GO containing many genes, whereas synaptic vesicle docking is a more niche term. This plot aids in identification of interesting terms and their associated symbols to explore further.

**Fig. 7:**
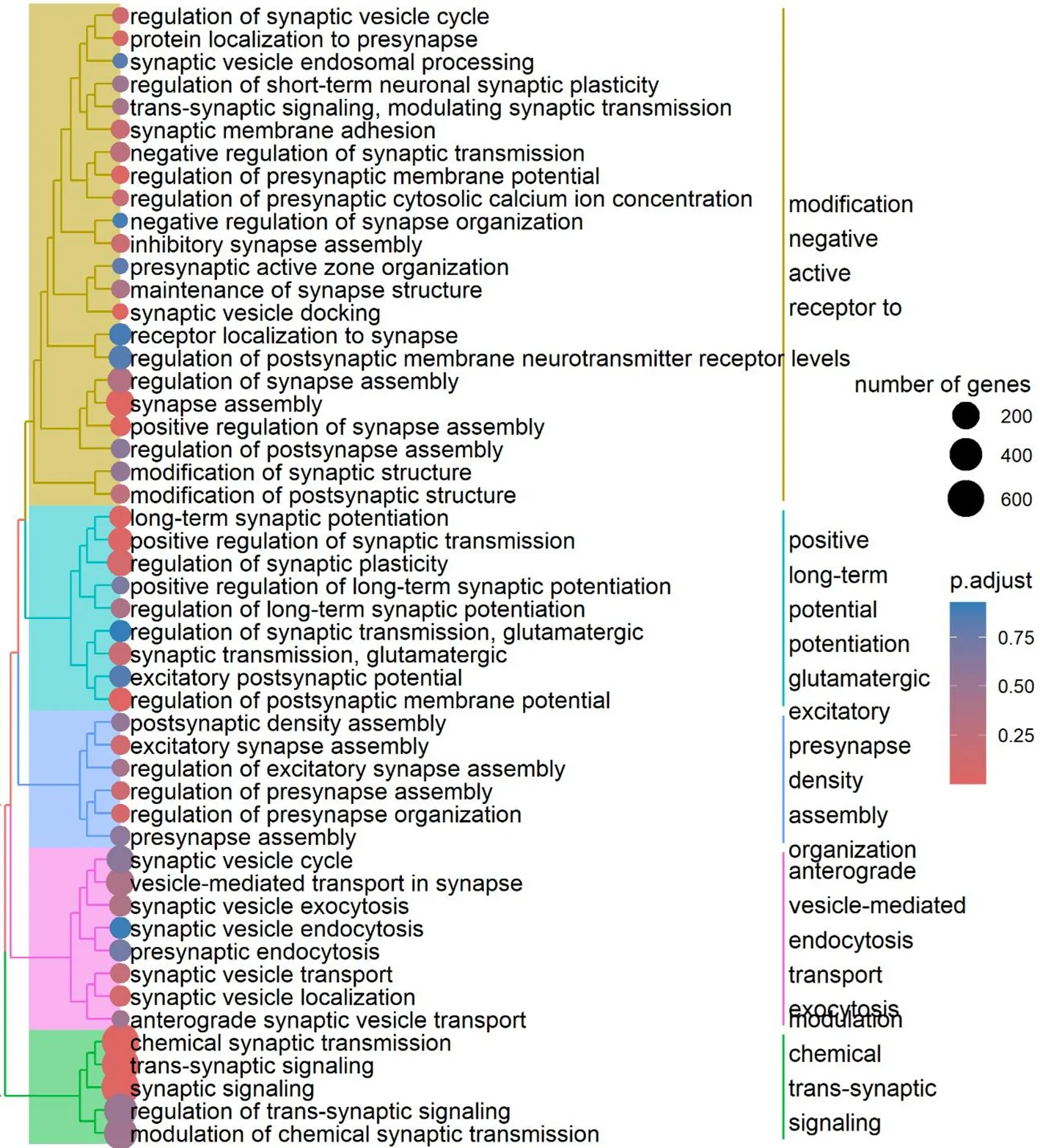
Tree plot with hierarchical clustering of top enriched terms by semantic similarity. Hierarchical clustering is shown colored. Terms are sized by the number of symbols in their gene set and colored by adjusted *p*-value. The words summarizing the semantic similarity are displayed on the right of the cluster lines.

The next step is made by plotting a term tree (Fig. 7) of the top enriched terms, made by hierarchically clustering term similarity. For the term tree, the number of terms and words for semantic similarity are user-set to visual inspection. Hierarchically clustering highlights functional modules and improves interpretability through reducing redundancy, it can inspire the user for further exploration through enrichment querying.

**Fig. 8:**
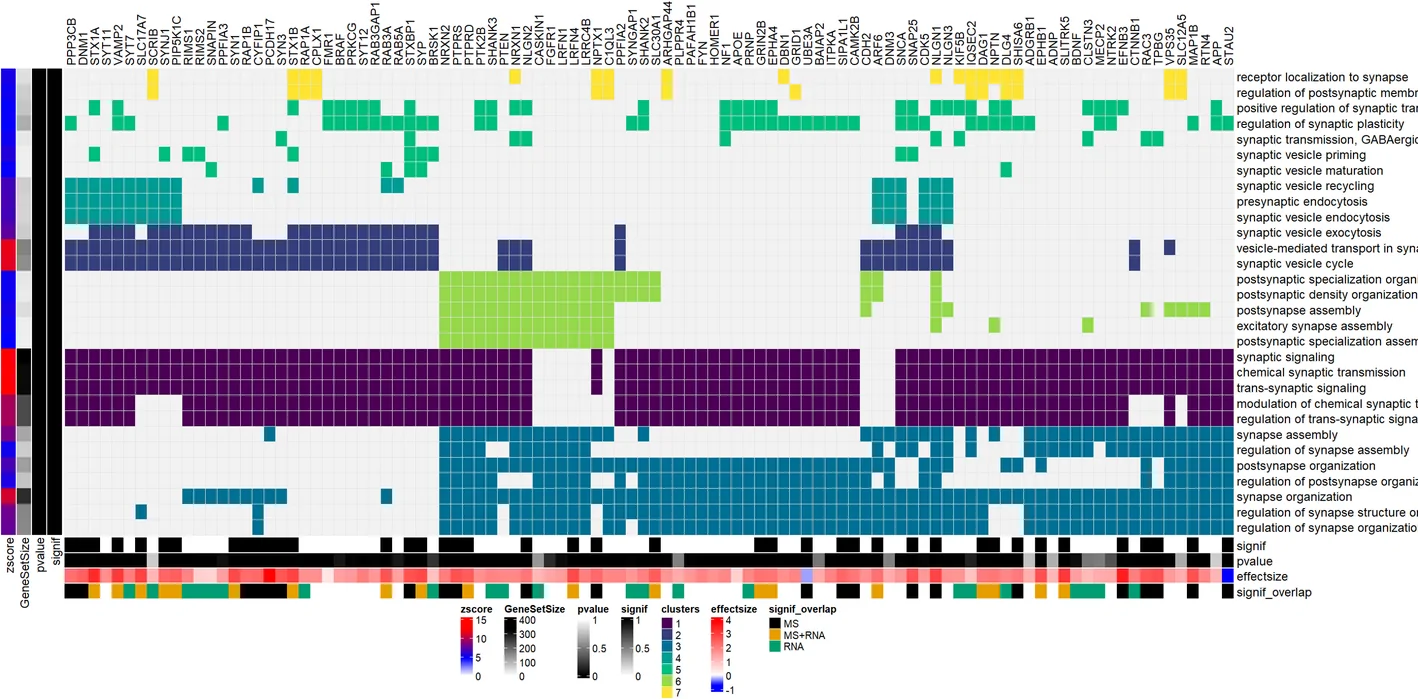
Interactive symbol-term overview heatmap of the queried enrichment result. Hierarchical clustering is done on symbols and terms, cells are colored by term cluster if a symbol is present in a corresponding term. Row annotations indicate the term-level metadata: gene set size, adjusted *p*-value, significance and *z*-score. Column annotations display symbol-level statistics: *p*-value, significance, effect size and overlap of significance between samples. Legend shows the discrete, color- or grayscale values for annotations.

GOATEA features an interactive term-symbol heatmap (Fig. 8) which includes metadata annotations to serve as an enrichment overview of the top enriched terms and top expressed symbols (here 30 and 75 respectively for visualization purposes). In the heatmap, a cell is colored if a symbol is present in a given term, with the color reflecting the assigned term cluster. Both terms and symbols are hierarchically clustered into a user-defined number of clusters by any method available from *stats::hclust()*, in this case, five clusters using the *ward.D* method. Row annotations indicate the term-level metadata: gene set size, adjusted *p*-value, significance and *z*-score. Column annotations display symbol-level statistics: *p*-value, significance, effect size and overlap of significance between samples. These annotations allow quick identification of functional biological modules by overlapping gene sets, or individual symbols that dominate enrichment signals, facilitating interpretation of how enriched terms and key symbols relate to each other. With dynamic querying of the enrichment result, relevant overviews can be focused on by dragging selection of a region of interest to generate a sub-heatmap. This focused view makes it possible to inspect a subset of terms and symbols in more detail, and to select symbols of interest for downstream exploration.

**Fig. 9:**
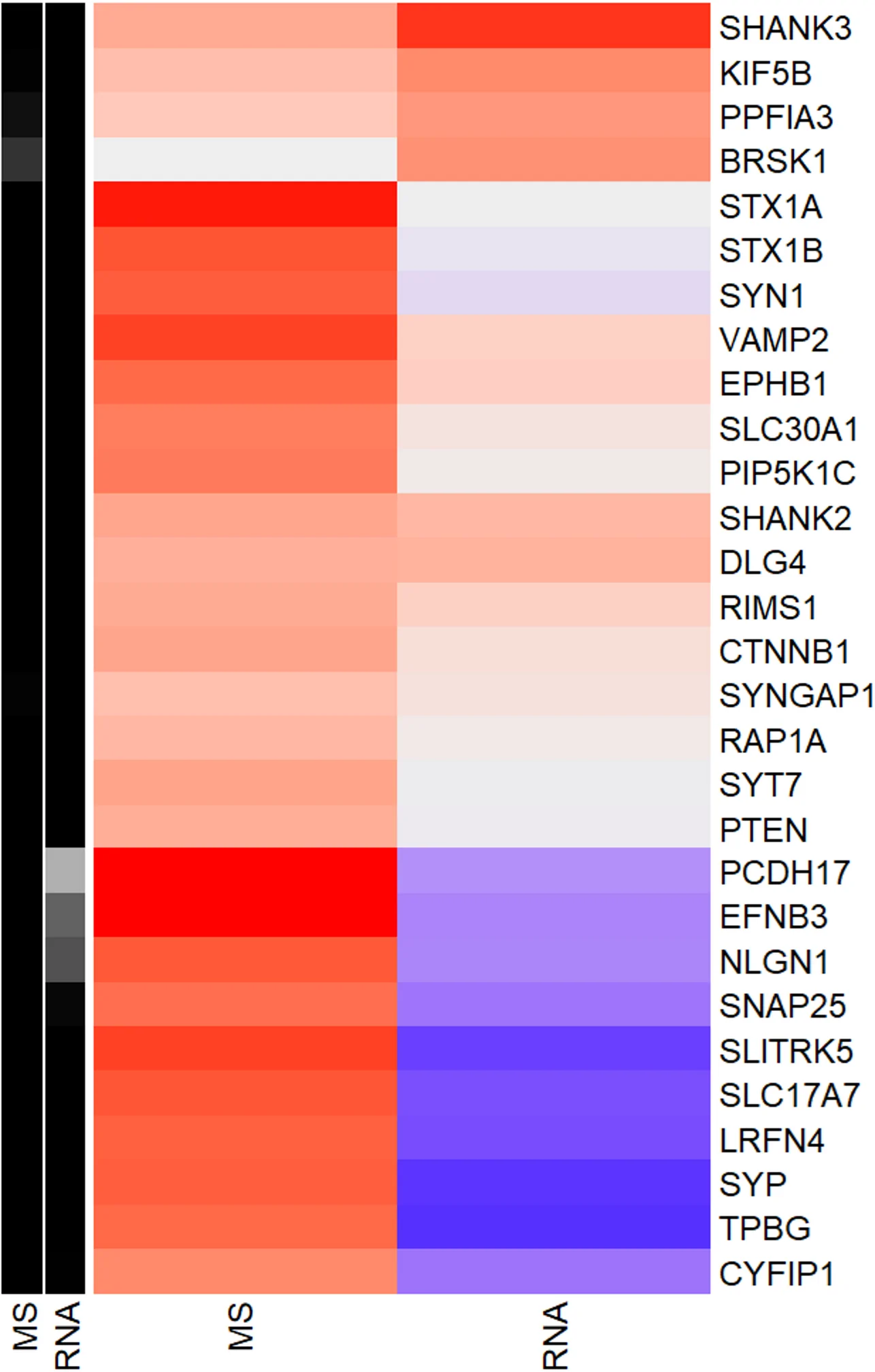
Interactive symbol-sample comparison subheatmap of user-selected symbols. Symbols and their hierarchical clustering are shown on the y-axis. Cells are colored by effect size and annotations are colored by *p*-value.

For instance, in the sample-symbol heatmap, the user-selected symbols are hierarchically clustered and used to compare p-values and effect sizes. Similar to the overview heatmap, a selection can be made for visual clarity by focusing on specific symbols, here the selected subset for generated subheatmap is shown (Fig. 9).

**Fig. 10:**
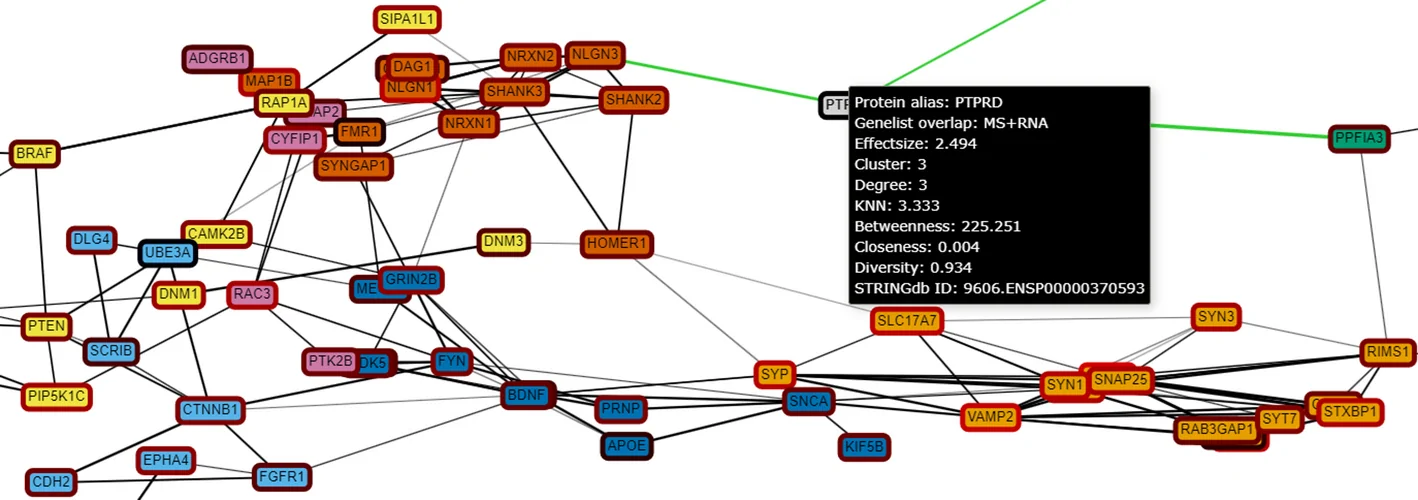
Interactive STRING database protein-protein interaction network subgraph of user-selected symbols. Protein-protein interaction strength is shown by edge opacity. Node outline color depicts a blue to red gradient for lower to higher effectsize respectively, Selected proteins are colored by the white surrounding box.

Running enrichment analyses with different scoring types, such as absolute effect size or effect size restricted to up- or down regulated symbols, allows integration of complementary perspectives on how expression changes contribute to enriched gene sets. As gene sets inherently lack topological and directional information between symbols, pathways and functional modules can be constructed using protein-protein interactions (PPI) in bottom-up fashion. A PPI network graph (cropped in Fig. 10) can be drawn from selected and additionally specified symbols, considered as proteins, for the user-set sample. Protein interaction data are temporarily downloaded from the STRING database with providing parameters for the database version and scoring threshold. Temporary data is converted to columnar Parquet format for efficient storage and processing by partially accessing the data without having to fully load it into memory. Then the data is formatted for input into igraph to use its functions to obtain statistics from drawn networks. From the igraph structure, a visNetwork interactive protein interaction graph is created for the user to explore. visNetwork uses igraph layouts for optimal network formation. The user can choose between the following layouts: ‘edge-weighted’ as default to represent STRING database scoring, ‘general’ to facilitate most datasets, ‘medium’ and ‘large’ as size-based layouts and ‘grid’, ‘circle’ and ‘gem’ for shape-based layouts. Once plotted, the user can highlight a specific symbol as well as highlight, filter or color by node and edge features to create and explore a network subgraph. Hovering edges and nodes show pop-up information and clicking the edge opens the STRING database site to detail that PPI. This network graph allows users to identify hub proteins, interpret whether selected proteins are tightly interconnected and assess whether distinct functional modules or clusters of proteins form. Such structural insights can reveal potential biological complexes, central regulators and bottom-up pathways in the dataset to guide future hypotheses.

#### Protein-protein interactions network-guided hypothesis generation

In this showcase, we applied GOATEA to a dataset investigating homeostatic synaptic plasticity to illustrate its analytical workflow. Here, we demonstrate identifying some relevant genes of interest as potential candidates to generate future hypotheses. As GOATEA can manage both genes and protein lists as input looking at overlapping symbols could provide potentially interesting findings. In the pre-enrichment UpSet overlap plot, 458 intersecting symbols were selected between the MS and RNA samples. These are then stringently filtered and explored in the network graph. The STRING database defined quality scores for PPI ranging from 1 to 1000, with our threshold set to 950 to retain qualitative PPI. Some isolated genes are found relevant to the topic, such as UBQLN1, encoding protein quality control protein ubiquilin-1 with a potentially neuroprotective role [39]. Similarly, NENF encodes a neuron-derived neurotrophic factor that may play a role in neuronal differentiation and development [40]. As well as MAP1A, a microtubule-associated protein, which has an essential role in synapse function [41–43]. Also, a relevant protein pair is identified. ADCY5-RAPGEF4, where adenylyl cyclases mediate G protein-coupled receptor signaling through cAMP. This activates Rap GEF that is in turn enriched at the postsynaptic density [44, 45]. There, Rap signaling remodels synapses and regulates neurotransmitter content, relevant to studying homeostatic synaptic plasticity. These candidates and associated proteins are potentially relevant for use as new starting points, building up bottom-up pathways and functional modules for exploration and hypothesis generation. With this, GOATEA shows efficient comparison of multiple contrasts in a single run, providing novel visualizations that take the user further than a summary of enriched gene sets. GOATEA highlights not only known synaptic plasticity genes but also less obvious, potentially overlooked candidates like UBQLN1 and NENF and functional interactions between ADCY5–RAPGEF4.

## Conclusions

Geneset Ordinal Association Test Enrichment Analysis (GOATEA) is a user-friendly R/Shiny application that extends the GOAT functional enrichment algorithm with novel interactive visualizations for exploratory data analysis. GOATEA addresses key limitations of existing enrichment tools by providing accessibility for users without programming expertise. Its interactive, gene- and network-based analysis provides contextual information for bottom-up pathway exploration and interpretation of enrichment patterns, enabling network-guided hypothesis generation. GOATEA showcases its capability for multi-contrast comparison and integration of matched modalities in multi-omics analysis as demonstrated on matched RNA sequencing and mass-spectrometry data. It streamlines enrichment workflows and empowers researchers to perform comprehensive, interpretable, and reproducible functional analyses. Ongoing development of GOATEA can be followed on GitHub.

## Supplementary Information

Below is the link to the electronic supplementary material.


Supplementary Material 1: ‘RNA and MS enrichment data’. The Additional Table file is called: “Additional File1.xlsx”. This file contains 2 tables, each on their own tab called the ‘enrichment_RNA’ and ‘enrichment_MS’ data, for reproducibility and exploration. If users want to download the raw data, instructions are provided in the “Datasets and databases" subsection of the “Implementation” section.



Supplementary Material 2: Qualitative comparison of GOATEA versus other gene set enrichment analysis tools. This file contains the single comparison table. “Bold highlighting indicates best in feature (column). GOAT = geneset ordinal association test. GSEA = gene set enrichment analysis. ORA = over-representation analysis. API = application programming interface. CLI = command line interface. pkg = package.”


## Data Availability

The data analysed during the current study are described and downloadable in the ‘Implementation’ section under the ‘Datasets and databases’ subsection. For ease of access, these data are available within the GOATEA GitHub repository: https://github.com/mauritsunkel/goatea and included in this article as Additional files. GOATEA package files and reference are also available on Bioconductor: https://bioconductor.org/packages/devel/bioc/html/goatea.html. Project name: GOATEA. Project home page: https://github.com/mauritsunkel/goatea. Operating system(s): Platform independent. Programming language: R. Other requirements: Rtools, software: see package DESCRIPTION file (all documented on GitHub). License: Apache 2.0. Any restrictions to use by non-academics: License-based.

## Abbreviations

DE: Differential expression
GO: Gene ontology
GSEA: Gene set enrichment analysis
GOAT: Geneset ordinal association test
GOATEA: Geneset ordinal association test enrichment analysis
GUI: Graphical user interface
MS: Mass spectrometry
ORA: Over-representation analysis
PPI: Protein-protein interactions
